# Polyionic polymers – heterogeneous media for metal nanoparticles as catalyst in Suzuki–Miyaura and Heck–Mizoroki reactions under flow conditions

**DOI:** 10.3762/bjoc.5.21

**Published:** 2009-05-08

**Authors:** Klaas Mennecke, Andreas Kirschning

**Affiliations:** 1 Institut für Organische Chemie and Zentrum für Biomolekulare Wirkstoffe (BMWZ), Leibniz Universität Hannover, Schneiderberg 1B, D-30167 Hannover, Germany

**Keywords:** Heck–Mizoroki reaction, heterogeneous catalysis, ion exchange resin, microreactor, monolith, palladium, Suzuki–Miyaura reaction

## Abstract

The preparation of monolithic polyionic supports which serve as efficient heterogeneous supports for palladium(0) nanoparticles is described. These functionalized polymers were incorporated inside a flow reactor and employed in Suzuki–Miyaura and Heck cross couplings under continuous flow conditions.

## Introduction

Functionalized solid supports like polymers loaded with homogeneous catalysts are well established in organic synthesis [1–4]. Simple purification of the products and easy recyclability of the catalysts are major advantages of heterogenization of transition metals. A major hurdle for industrial applications of heterogenized homogeneous metal catalyst is associated with keeping metal leaching down to a minimum. Immobilization can be regarded as one enabling technique in organic chemistry [5–6] that in conjunction with continuous flow processes creates an ideal setup for an automated solution-phase synthesis. Furthermore, this combination of enabling techniques has great potential in the production of fine chemicals [7–8].

In continuation of our efforts in developing immobilization concepts for reagents and catalysts including transition metals on solid phases inside monolithic flow reactors [9–18] we describe the preparation of palladium nanoparticles loaded on polyionic polymers and their use under continuous flow conditions in various C-C-cross-coupling reactions [19–22].

## Results and Discussion

### Preparation of the catalyst

**Scheme 1:**
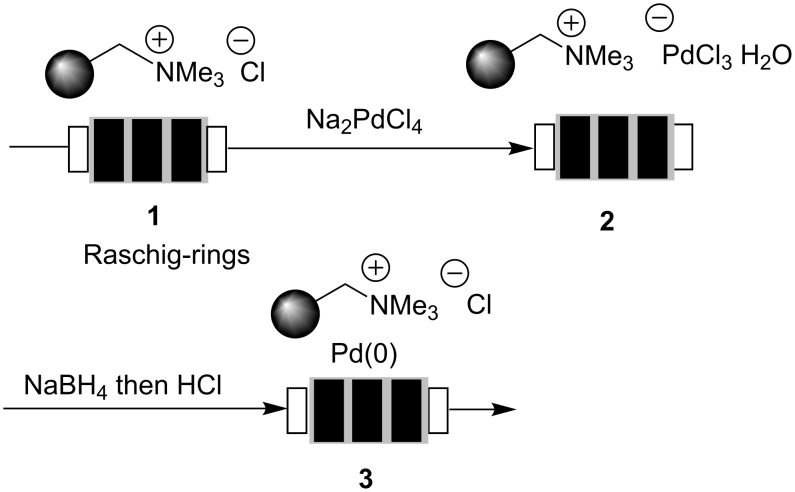
Preparation of Pd(0) nanoparticles inside flow reactors.

**Figure 1:**
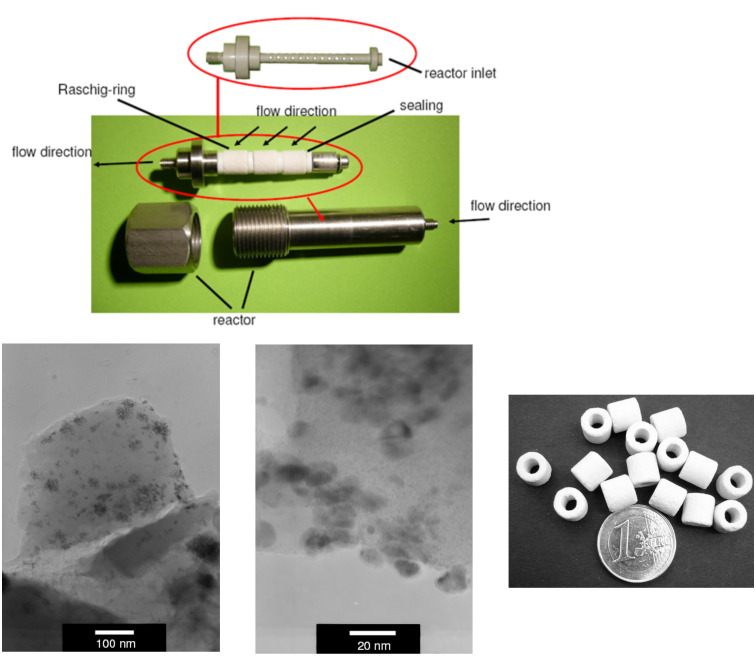
Top: Reactor (1–2 mL dead volume) with functionalized Raschig-rings; bottom: TEM-micrographs of Pd(0) nanoparticles on optimized polyionic gel (left and central) and Raschig-rings (right).

Recently, we reported on the preparation of a monolithic polymeric material incorporated inside megaporous glass shaped Raschig-rings [11–18,23–24]. The polymeric phase was created by radical precipitation polymerization of styrene, vinylbenzyl chloride and divinylbenzene as monomers and consists of very small bead-like particles (0.2–2 µm) which are connected through polymeric bridges. As a result an extended monolithic polymeric phase inside a glass monolith is created. Incorporation of the resin inside a porous glass has the advantage that the resin can only swell inside the glass while the glass monolith provides a stable rod-like shape inside the microreactor. The Merrifield-type resin was aminated to yield polyionic support **1**. This polymer serves as an anchor to leave the metal species (sodium tetrachloropalladate; Na_2_PdCl_4_) in close proximity to the ammonium group by means of ion exchange (Scheme 1). In the following, the active Pd particle is generated upon reduction with a solution of sodium borohydride. A particular benefit of the resulting solid support is the stabilization of the generated nanoparticles by the polymer-bound ammonium species [23–29]. These functionalized composite Raschig-rings are incorporated inside the flow microreactor which has a dead volume of about 1–2 mL (Figure 1) [30]. We could show that the palladium clusters are composed of palladium nanoparticles. Particle sizes highly depend on the monomer composition of the polyionic support [4-vinylbenzyl chloride (VBC), divinylbenzene (DVB), styrene]. Depending on the particle diameter a large impact on their catalytic performance (batch vs. flow; conventional vs. microwave heating) was noted [24]. In this context these materials have clear advantages over Pd(0) on charcoal because the latter cannot be further optimized with respect to the mode of application [31–33]. In the present study, our highly optimized composite material was chosen (5.3% DVB crosslinker and a 1:1 mixture of VBC/styrene) doped with nanoparticles (7–10 nm in size and a palladium content of 0.03 weight% Pd on polyionic polymer).

### Suzuki–Miyaura cross coupling reactions

In our earlier work we showed that these materials are well suited for transfer hydrogenations under flow conditions [23–24]. Recently, the Suzuki–Miyaura reaction and other palladium catalyzed reactions have emerged as industrially very desirable processes and miniflow fixed bed reactors loaded with Pd(0) nanoparticles should be well suited to perform these C-C coupling reactions [34]. A particular challenge for utilizing the Suzuki–Miyaura reaction in flow devices is the quest for truly homogeneous reaction conditions in order to prevent clogging of the irregular microchannels. We chose the coupling of 4′-bromoacetophenone and phenylboronic acid as model reaction for optimizing the process and found that 85% conversion could be achieved within 10 min at 95 °C in DMF/water 10/1 with 2.5 mol% of catalyst **3** using CsF as base. The reaction was performed in a cyclic mode with a flow rate of 2 mL/min. Single pass experiments with flow rates between 0.1 and 1 mL/min did not result in improved results, so that we commonly operated the system as a closed loop reactor in the following.

Under these optimized conditions several examples of successful cross coupling reactions were achieved that are listed in Table 1. We included combinations of electron rich and electron deficient aryl bromides with functionalized boronic acids and yields of coupling products were commonly good to excellent. Aryl chlorides did not react with catalyst **3** under flow conditions.

**Table 1:** Suzuki–Miyaura reactions catalyzed by Pd nanoparticles **3** inside flow reactors.

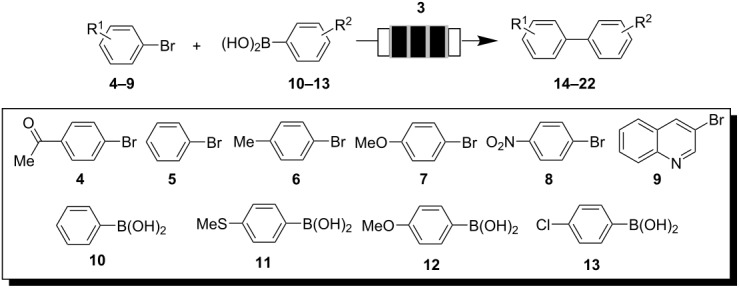				
aryl bromide	boronic acid	product	time [h]	yield [%]^a^

**4**	**10**	**14**	1	85
**5**	**10**	**15**	5.5	85
**6**	**10**	**16**	5.5	60
**7**	**10**	**17**	3.5	75
**8**	**10**	**18**	4.5	99
**9**	**10**	**19**	24	86
**4**	**11**	**20**	1	81
**4**	**12**	**21**	2.5	89
**4**	**13**	**22**	2	99

^a^Isolated yield of pure product.

**Figure 2:**
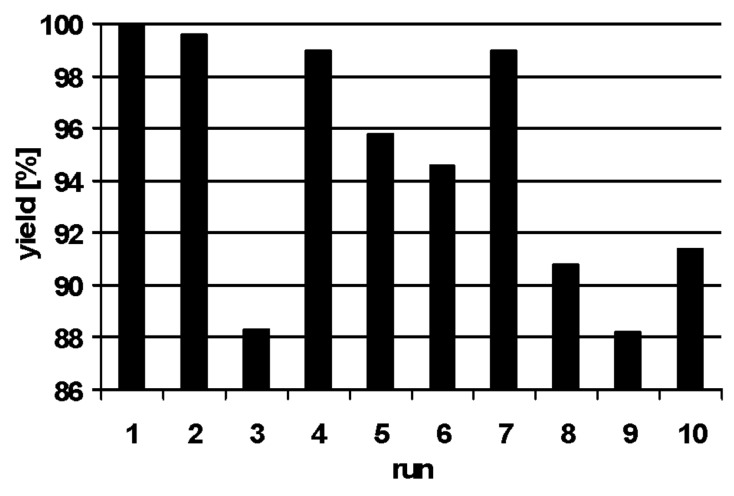
Repeated Suzuki reaction of 4-bromotoluene (**6**) with phenylboronic acid (**10**) under flow conditions. Deviations may result from work up as only isolated yields are presented.

To fully explore the potential of polyionic gel **3** its reusability was investigated next. The Suzuki reaction of 4-bromotoluene (**6**) with phenylboronic acid (**10**) served as model reaction. After each reaction the continuous flow reactor was regenerated by pumping a solution of DMF/water (10:1, 40 mL) through the reactor before the next run was initiated (Figure 2). The palladium particles inside the flow reactor showed excellent stability without loss of activity after the tenth run. Palladium leaching was determined to be about 0.7 ppm for each run. This very low value for leaching corresponds to the leaching determined for transfer hydrogenations with this catalytic flow system using cyclohexene as hydrogen source and solvent [23–24].

### Heck–Mizoroki reactions

One other very important cross coupling reaction that bears industrial relevance is the Heck–Mizoroki reaction. We were able to perform C-C coupling reaction under flow conditions with aryl iodides **23**–**28** using catalyst **3** (Table 2). Optimization of the conditions for our monolithic flow reactor was conducted with 4′-iodoacetophenone (**23**) and styrene (**29**) as coupling partners. With *n*-butylamine as base and 2.5 mol% catalyst **3** in DMF at 120 °C and a flow rate of 2 mL/min it was possible to achieve full conversion with complete *E*-selectivity within 30 min. Formation of by-products resulting from homocoupling was not observed. When 4′-iodoacetophenone (**23**) was exchanged with 4′-bromoacetophenone coupling with styrene yielded Heck-product **30** in only 35%.

**Table 2:** Heck–Mizoroki reactions catalyzed by Pd nanoparticles **3** inside flow reactors.

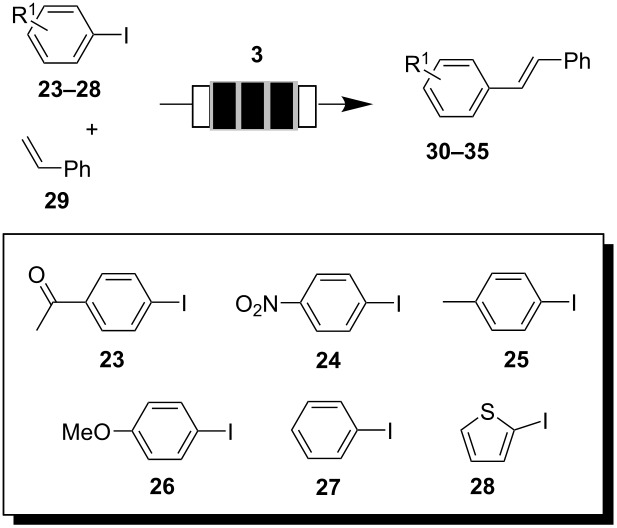			
aryl iodide	product	time [h]	yield [%]^a^

**23**	**30**	0.5	99
**24**	**31**	24	99
**25**	**32**	19	93
**26**	**33**	3	77
**27**	**34**	24	99
**28**	**35**	4	99

^a^Isolated yield of pure product.

In order to generalize the reaction protocol different aryl iodides were coupled with styrene. In all cases, the C-C coupling products were formed within 0.5 to 24 h in very good yield with excellent stereocontrol (see Table 2). Palladium leaching was determined to be 0.04% for each run based on the catalyst used initially, which is an exceptionally low value in view of the fact that DMF a well coordinating solvent is employed [35].

Even commercially available and widely employed catalysts that are based on encapsulated Pd particles such as PdEnCat [35] show a similarly low degree of leaching in DMF to our Pd nanoparticles [36]. With reference to the fundamental work by Reetz and de Vries the ionic environment on the polymer phase that is located in very close vicinity to the palladium nanoparticles has very likely to be made responsible for the stabilization of the nanoparticles which results in low degree of leaching [25–29].

**Figure 3:**
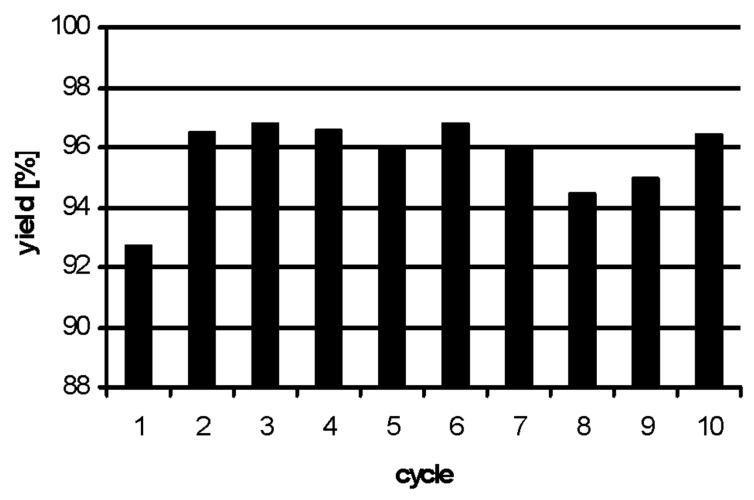
Repeated Heck–Mizoroki reaction of 4′-iodoacetophenone (**23**) with styrene (**29**) under flow conditions.

It has to be noted that like many other heterogenized Pd sources [15–16,36–41] these polyionic gels very likely also serve as reservoirs for Pd nanoclusters that are released into solution at very low concentrations. With respect to transfer hydrogenations using precatalyst **3** we recently conducted a thorough study on the principal question whether **3** serves as a Pd reservoir [23]. As was first demonstrated by Reetz [25–28] and de Vries [29] these clusters exert pronounced catalytic activity in solution at very low concentrations. This view is further supported by the fact that the catalytic species operating in the present case is able to promote Suzuki–Miyaura cross coupling reactions with aryl bromides while aryl chlorides are not good substrates under these standard conditions. This observation has been noted in many examples of heterogenized palladium salts or complexes [34]. Likewise, these species are commonly not reactive enough to promote the Heck–Mizoroki reaction with aryl bromides. It should be noted that under supercritical or high pressure/high temperature conditions aryl chlorides (for Suzuki–Miyaura reactions) or aryl bromides (for Heck–Mizoroki reactions) may very well serve as substrates for this kind of palladium species.

## Conclusion

In summary, we demonstrated that polyionic gel **3** is a well suited ion exchange resin for the generation of metal nanoparticles. The ionic nature of the resin has a positive impact on the stabilization of the Pd species which results in extended use for Suzuki–Miyaura cross coupling reactions as well as Heck reactions without substantial reduction of activity even when solvents such as DMF are employed and therefore leads to a minimum degree of leaching. The ease of preparation and the properties of polyionic catalyst **3** make it an attractive catalytic monolith for industrially relevant continuous flow processes particularly when employed in combination with a scavenger column for removing traces of soluble Pd species.

## Supporting Information

File 1 Experimental

## Note added in proof

In a detailed study McQuade and coworker demonstrated that PdEnCat 30 also behaves as heterogeneous sources for soluble, catalytically active species during the course of Heck and Suzuki couplings. They note a solution-phase contribution for catalysis and determined leaching up to 46% in DMF as solvent which is significantly higher compared to our polyionic gels: Broadwater, S. J.; McQuade, D. T. *J. Org. Chem.* **2006**, *71*, 2131–2134.
