# Supplementary material for: Polyionic polymers – heterogeneous media for metal nanoparticles as catalyst in Suzuki–Miyaura and Heck–Mizoroki reactions under flow conditions
Source: Beilstein J Org Chem. 2009 May 8;5:21. doi: 10.3762/bjoc.5.21 (PMC2707061; doi:10.3762/bjoc.5.21)
Supplement: File 1 — Experimental [file Beilstein_J_Org_Chem-05-21-s001.doc]

SUPPORTING INFORMATION

Polyionic polymers - heterogeneous media for metal nanoparticles as catalyst in Suzuki–Miyaura and Heck–Mizoroki reactions under flow conditions

Klaas Mennecke and Andreas Kirschning*

Address: Institut für Organische Chemie and Zentrum für Biomolekulare Wirkstoffe (BMWZ), Leibniz Universität Hannover, Schneiderberg 1B, D-30167 Hannover, Germany

Email: Andreas Kirschning* - andreas.kirschning@oci.uni-hannover.de

* Corresponding author

Experimental

NMR spectra were recorded with a Bruker DPX-400 spectrometer at 400 MHz (^1^H NMR) and at 125 MHz (^13^C NMR) in CDCl_3_. Mass spectra (EI) were obtained at 70 eV with a type VG Autospec apparatus (Micromass). GC analyses were conducted using a Hewlett-Packard HP 6890 Series GC System equipped with a SE-54 capillary column (25 m, Macherey-Nagel) and a FID detector 19231 D/E. Melting points were determined in open glass capillaries with a Gallenkamp apparatus and are uncorrected. Analytical thin-layer chromatography was performed using precoated silica gel 60 F_254_ plates (Merck, Darmstadt), and the spots were visualised with UV light at 254 nm or by staining with H_2_SO_4_/4-methoxybenzaldehyde in ethanol. Flash column chromatography was performed on Merck silica gel 60 (230–400 mesh). TEM measurements were conducted on a Philips CM 200 microscope at 200 kV. High resolution micrographs were collected on a JEOL JEM-2100F-UHR (providing a point-resolution better than 0.19 nm at 200 kV). Samples were collected after crushing composite Raschig-rings into fine powder, dispersion of the material in a volatile liquid (ethanol) and finally subjecting it to a fine mesh carbon net which is electron transparent. Ion exchange measurements of palladate were achieved by UV-vis spectroscopy of the palladate solution using a Hach DR 4000V Spectrophotometer (430 m wavelength). Commercially available reagents and dry solvents (DMF) were used as received. Most compounds prepared were isolated after filtration and removal of the solvent. Pure homogeneous product samples were collected after flash chromatography and purity was analyzed by TLC, GC and NMR analyses. Characterization was achieved by appropriate spectroscopic methods (^1^H and ^13^C NMR, MS). Additionally, for known compounds, a comparison of physical (e.g., melting points) and spectroscopic data with those in the literature was made.

**Preparation of the polymeric phase**

The polymeric sample was prepared by polymerization of 4-vinylbenzyl chloride (VBC, 90% purity, 47.3 mol%) and styrene (99% purity, 47.3 mol%) in the presence of divinylbenzene (DVB, 65% purity, 5.3 mol%) as cross linking monomer using precipitation polymerization inside a fractal porous Raschig-ring. The polymeric sample obtained by precipitation polymerization was functionalized in the following by amination with triethylamine in toluene which afforded the corresponding polymer functionalized with quaternary ammonium chloride sites. The polymer content of the composite material was determined to be about 8 weight% and the capacity was analysed to be 0.284 mmol/g.

**Palladium loading procedure**

Loading was carried out by incorporating five Raschig-rings inside a stainless steel PASSflow reactor. The palladate ion exchange was achieved within 2 h by circulating an aqueous solution (0.028 M; 1:1 ratio ion exchange capacity and palladate) of Na_2_Cl_4_Pd∙3H_2_O through the reactor. In the following, the reactor was flushed for one hour with distilled water at a flow rate of 5 mL/min before a solution of borohydride in water (1.5 g, 39.65 mmol; 200 ml) was pumped through the PASSflow reactor at a flow rate of 10 mL/min. The loading was terminated after three washing steps: a) distilled water, 1 h, 5 mL/min; b) 100 mL 2 M HCl, 9 mL/min and c) distilled water, 9 mL/min until the pH at the outlet was determined to be 7. After drying the Pd content of the functionalized polymer-glass composite rings was analyzed and was determined to be 0.263 mmol (average out of 10 determinations) Pd/g ring.

**Analytical determination of Pd leaching.**

All samples were digested with 4 mL sub boiled HNO_3_ and 1 mL H_2_O_2_ (p.a., Merck) and afterwards diluted with water. The analysis of Pd content was carried out in an inductively coupled plasma mass spectrometer (ICP-MS) Thermo X7 ICP-MS (Thermo Electron GmbH, Dreieich, Germany, www.thermo.com). This quadrupol instrument had a power of 1200 W throughout the measurement. The gas flow rates were 1.0 mL/min (Nebuliser), 1.2 mL/min (Auxiliary) and 13 mL/min (Cool) argon 4.8, respectively. ^105^Pd, ^106^Pd, ^108^Pd and ^110^Pd were the isotopes monitored. Quantification was performed with liquid Pd standards from 0.01–50 µg/L averaging the concentrations obtained for all 4 isotopes. For drift corrections during the measurements the standard with the concentration of 1 µg/L was interspaced after every second sample measurement. Under the conditions used the limit of detection for palladium was 1 ng/L.

**Suzuki–Miyaura reaction under continuous flow conditions**.

A solution of aryl bromide (1 mmol), arylboronic acid (1.5 mmol) and cesium fluoride (364 mg, 2.4 mmol) in DMF/water (10:1, 5 mL) was circulated (2 mL/min) through the microreactor filled with one Raschig-ring (0.025 mmol Pd, 2.5 mol% Pd) at 95 °C. After completion of the reaction (GC monitoring), the micro reactor was washed with DMF/water 10:1 (40 mL). The mixture was diluted with water and extracted with EtOAc (3x). The combined organic phases were dried (MgSO_4_), concentrated in vacuum, and the product was purified by flash column chromatography (mixtures of ethyl acetate / petroleum ether) on silica gel. The reactor was ready to be used for the next experiment.

**Analytical data of Suzuki coupling products**

**4-Acetyl-1,1'-biphenyl (14)**

The coupling product was isolated in 85% yield.

Colorless crystals: mp = 120 °C (Lit. [1]: 118–120 °C)

^1^H NMR (CDCl_3_): δ = 2.64 (s, 3H, C*H*_3_), 7.41 (dd, *J =* 7.3, 7.3 Hz, 1H, H_ar_), 7.48 (dd, *J =* 7.8, 7.3 Hz, 2H, H_ar_), 7.63 (d, *J =* 7.8 Hz, 2H, H_ar_), 7.69 (d, *J =* 8.5 Hz, 2H, H_ar_), 8.04 (d, *J =* 8.5 Hz, 2H, H_ar_).

^13^C NMR (CDCl_3_): δ: 26.8, 127.3, 127.4, 128.4, 129.0, 129.1, 136.0, 140.0, 145.9, 197.9.

HR-MS: found 196.0889; calcd for C_14_H_12_O 196.0888.

**1,1'-Biphenyl (15)**

The coupling product was isolated in 85% yield.

Colorless crystals: mp = 71 °C. (Lit. [2] 70.5 °C)

^1^H NMR (CDCl_3_): δ = 7.6 (d, *J =* 8 Hz, 4H, H_ar_), 7.47–7.42 (m, 4H, H_ar_), 7.37–7.33 (m, 2H, H_ar_).

^13^C NMR (CDCl_3_): δ = 141.4, 128.9, 127.4, 127.3

MS (EI): found 154; calcd for C_12_H_10_: 154.

**4-Methyl-1,1'-biphenyl** **(16)**

The coupling product was isolated in 60% yield.

Colorless crystals: mp = 48 °C (Lit. [3] 47–48 °C)

^1^H NMR (CDCl_3_): δ = 7.58 (d, *J =* 7.2 Hz, 2H, 2'-H), 7.5 (d, *J =* 8.2 Hz, 2H, 2-H), 7.43 (m, 2H, 3'-H), 7.32 (m, 1H, 4'-H), 7.25 (d, *J =* 8.2 Hz, 2H, 3-H), 2.4 (s, 3H, Me).

^13^C NMR (CDCl_3_): δ = 141.3, 138.5, 137.2, 129.6, 128.8, 127.14, 127.12, 127.11, 21.2.

MS (EI): found 168; calcd for C_13_H_12_ 168.

**4-Methoxy-1,1'-biphenyl** **(17)**

The coupling product was isolated in 75% yield.

Colorless crystals: mp = 89 °C (Lit. [4] 84–86 °C)

^1^H NMR (CDCl_3_): δ = 7.55 (d, *J =* 7.5 Hz, 2H, 2'-H), 7.53 (d, *J =* 8.8 Hz, 2H, 2-H), 7.42 (dd, *J =* 7.5, 7.5 Hz, 2H, 3'-H), 7.32–7.28 (m, 1H, 4'-H), 6.98 (d, *J =* 8.8 Hz, 2H, 3-H), 3.85 (s, 3H, O*Me*).

^13^C NMR (CDCl_3_): δ = 159.3, 140.9, 133.9, 128.9, 128.3, 126.9, 126.8, 114.3, 55.5.

MS (EI): found 184; calcd for C_13_H_12_O 184.

**4-Nitrobiphenyl (18)**

The coupling product was isolated in 99% yield.

Colorless crystals: mp = 114 °C (Lit. [5,6]: 114 °C)

^1^H NMR (CDCl_3_): δ = 8.31–7.82 (m, 2H, H_ar_), 7.72 (m, 2H, H_ar_), 7.64–7.62 (m, 2H, H_ar_), 7.52–7.43 (m, 3H, H_ar_).

^13^C NMR (CDCl_3_): δ = 147.6, 138.9, 129.3, 129, 128.5, 127.9, 124.2.

MS (EI): found 199; calcd for C_12_H_9_NO_2_ 199.

**3-Phenylquinoline (19)**

The coupling product was isolated in 86% yield.

Pale yellow crystals: mp = 52 °C (Lit. [7] 52 °C)

^1^H NMR (CDCl_3_): δ = 9.19 (s, 1H, H_ar_), 8.26 (s, 1H, H_ar_), 8.16 (d, *J =* 8.2 Hz, 1H, H_ar_), 7.84 (d, *J =* 8.2 Hz, 1H,), 7.73 (m, 1H, H_ar_), 7.69 (d, *J =* 8.2 Hz, 2H, H_ar_), 7.56 (d, *J =* 7.8 Hz, 1H, H_ar_), 7.5 (dd, *J =* 8.2, 7.16 Hz, 2H, H_ar_), 7.42 (dd, *J =* 7.2, 7.5 Hz, 1H, H_ar_).

^13^C NMR (CDCl_3_): δ = 149.9, 147.4, 137.9, 133.9, 133.2, 129.7, 129.4, 129.3, 129.2, 128.1, 128, 127.5, 127.

MS (EI): found 204; calcd for C_15_H_11_N 204.

**4-Acetyl-4'-(methylthio)-1,1'-biphenyl (20)**

The coupling product was isolated in 81% yield.

Colorless crystals: mp = 185–186 °C (Lit. [8] 182–183.5 °C)

^1^H NMR (CDCl_3_): δ = 8.02 (d, *J =* 8.53 Hz, 2H, H_ar_), 7.66 (d, *J =* 8.53 Hz, 2H, H_ar_), 7.56 (d, *J =* 8.53 Hz, 2H, H_ar_), 7.34 (d, *J =* 8.53, 2H, H_ar_), 2.63 (s, 3H, C*H*_3_C=O), 2.53 (s, 3H, SC*H*_3_).

^13^C NMR (CDCl_3_): δ = 197.8, 145.2, 139.3, 136.6, 135.9, 129.1, 127.7, 126.96, 126.9, 26.8, 15.8.

MS (EI): found: 242; calcd for C_15_H_14_OS: 242.

**4-Acetyl-4'-methoxy-1,1'-biphenyl (21)**

The coupling product was isolated in 89% yield.

Colorless crystals: mp = 154 °C (Lit. [9] 153–154 °C)

^1^H NMR (CDCl_3_): δ = 8.01 (d, *J =* 8.2 Hz, 2H, H_ar_), 7.64 (d, *J =* 8.2 Hz, 2H, H_ar_), 7.58 (d, *J =* 8.9 Hz, 2H, H_ar_), 7 (d, *J =* 8.9 Hz, 2H, H_ar_), 3.86 (s, 3H, C*H*_3_O), 2.63 (s, 3H, C*H*_3_C=O).

^13^C NMR (CDCl_3_): δ = 197.9, 160.1, 145.5, 135.4, 132.4, 129.1, 128.5, 126.8, 114.6, 55.5, 26.8.

MS (EI): found: 226; calcd for C_15_H_14_O_2_: 226.

**4-Acetyl-4'-chloro-1,1'-biphenyl (22)**

The coupling product was isolated in 99% yield.

Colorless crystals: mp = 102–103 °C (Lit. [9] 103–104 °C)

^1^H NMR (CDCl_3_): δ = 8.03 (d, *J =* 8.53 Hz, 2H, H_ar_), 7.65 (d, *J =* 8.53 Hz, 2H, H_ar_), 7.56 (d, *J =* 8.53 Hz, 2H, H_ar_), 7.44 (d, *J =* 8.53 Hz, 2H, H_ar_), 2.64 (s, 3H, C*H*_3_).

^13^C NMR (CDCl_3_): δ = 197, 144.6, 138.5, 136.3, 134.6, 129.3, 129.2, 128.7, 127.2, 26.8.

MS (EI): found: 230; calcd for C_14_H_11_ClO: 230.

**Heck reaction under continuous flow conditions**.

A solution of aryl halide (1 mmol), alkene (3 mmol) and tri-*n*-butylamine (556 mg, 3 mmol) in dry DMF (3 ml) was circulated at 120 °C through the reactor (flow rate: 2 mL/min) charged with one Raschig-ring bearing catalyst **3** (0.263 mmol Pd/g ring, 2.5 mol% Pd). After completion of the reaction (GC-monitoring), the reactor was rinsed with DMF (40 ml). The solution was diluted with water (80 ml), extracted with EtOAc (3 x 50 ml) and dried (MgSO_4_). After concentration in vacuum, the products were commonly sufficiently pure. Final purification was possible by flash column chromatography (mixtures of ethyl acetate/petroleum ether) on silica gel. The reactor was ready to be used for the next experiment.

**Analytical data of Heck coupling products**

**1-(4-Styrylphenyl)ethanone (30)**

The coupling product was isolated in 99% yield.

Colorless crystals: mp = 134–137 °C (Lit. [4] 134–137 °C)

^1^H NMR (CDCl_3_): δ = 8 (d, *J =* 7.4 Hz, 2H, H_ar_), 7.65 (d, *J =* 8 Hz, 2H, H_ar_), 7.59 (d, *J =* 7.4 Hz, 2H, H_ar_), 7.32 (m, 5H), 2.62 (s, 3H, Me).

^13^C NMR (CDCl_3_): δ = 197.6, 142.1, 136.8, 136, 131.5, 129, 128.9, 128.4, 127.5, 126.9, 126.6, 26.7.

MS (EI): found: 222; calcd for C_16_H_14_O: 222.

***trans*-4-Nitrostilbene (31)**

The coupling product was isolated in 99% yield.

Colorless crystals: mp = 154 °C (Lit. [10] 155 °C)

^1^H NMR (CDCl_3_): δ = 8.22 (d, *J =* 8.6 Hz, 2H, H_ar_), 7.64 (d, *J =* 8.6 Hz, 2H, H_ar_), 7.55 (m, 2H, H_ar_), 7.43–7.31 (m, 3H, H_ar_), 7.27 (d, *J =* 16 Hz, 1H, C*H*ArC*H*Ar), 7.14 (*J =* 16 Hz, 1H, C*H*ArC*H*Ar).

^13^C NMR (CDCl_3_): δ = 146.9, 136.3, 134.1, 133.5, 129.05, 129, 127.2, 127, 126.4, 124.3.

MS (EI): found: 199; calcd for C_12_H_9_NO_2_: 199.

***trans*-4-Methylstilbene (32)**

The coupling product was isolated in 93% yield.

Colorless crystals: mp = 120 °C (Lit. [4] 112–115 °C)

^1^H NMR (CDCl_3_): δ = 7.5 (d, *J =* 7.16 Hz, 2H, H_ar_), 7.42 (d, *J =* 8.2 Hz, 2H, H_ar_), 7.35 (dd, *J =* 7.5, 7.16 Hz, 2H, H_ar_), 7.25 (dd, *J =* 7.5, 7.3 Hz, 1H, H_ar_), 7.17 (d, *J =* 8.2 Hz, 2H, H_ar_), 7.1 (d, *J =* 16.4 Hz, 1H, C*H*ArC*H*Ar), 7.05 (d, *J =* 16.4 Hz, 1H, C*H*ArC*H*Ar), 2.36 (s, 3H, Me).

^13^C NMR (CDCl_3_): δ = 137.671, 137.665, 134.7, 129.5, 128.8, 128.77, 127.8, 127.5, 126.57, 126.54, 21.4.

MS (EI): found: 194; calcd for C_15_H_14_: 194.

***trans*-4-Methoxystilbene (33)**

The coupling product was isolated in 77% yield.

Colorless crystals: mp = 136 °C (Lit. [11] 134 °C)

^1^H NMR (CDCl_3_): δ = 7.49 (d, *J =* 7.16 Hz, 2H, H_ar_), 7.45 (d, *J =* 8.87 Hz, 2H, H_ar_), 7.35 (dd, *J =* 7.68, 7.16 Hz, 2H, H_ar_), 7.25–7.22 (m, 1H, H_ar_), 7.07 (d, *J =* 16.4 Hz, 1H, C*H*ArC*H*Ar), 6.97 (d, *J =* 16.4 Hz, 1H, C*H*ArC*H*Ar), 6.9 (d, *J =* 8.87 Hz, 2H, H_ar_), 3.84 (s, 3H, O*Me*).

^13^C NMR (CDCl_3_): δ = 159.5, 137.8, 130.3, 128.8, 128.4, 127.9, 127.4, 126.8, 126.4, 114.3, 55.5.

MS (EI): found: 210; calcd for C_15_H_14_O: 210.

***trans*-Stilbene (34)**

The coupling product was isolated in 99% yield.

Colorless crystals: mp = 124 °C (Lit. [12] 124 °C)

^1^H NMR (CDCl_3_): δ = 7.56 (m, 4H, H_ar_), 7.36–7.44 (m, 4H, H_ar_), 7.25–7.33 (m, 2H, H_ar_), 7.15 (s, 2H, C*H*ArC*H*Ar).

^13^C NMR (CDCl_3_): δ = 137.5, 128.8, 127.8, 126.6.

MS (EI): found: 180; calcd for C_14_H_12_: 180.

***trans*-2-Styrylthiophene (35)**

The coupling product was isolated in 99% yield.

Colorless crystals: mp = 112 °C (Lit. [13] 109–110 °C)

^1^H NMR (CDCl_3_): δ = 7.47 (d, *J =* 7.5 Hz, 2H, H_ar_), 7.34 (dd, *J =* 7.68, 7.5 Hz, 2H, H_ar_), 7.27–7.25 (m, 1H, H_ar_), 7.23 (d, *J =*16 Hz, 1H, C*H*ArC*H*Ar), 7.19 (d, *J =* 5 Hz, 1H, H_ar_), 7.0 (d, *J =* 3.4 Hz, 1H, H_ar_), 7 (dd, *J =* 5, 3.4 Hz, 1H, H_ar_), 6.93 (d, *J =* 16 Hz, 1H, C*H*ArC*H*Ar).

^13^C NMR (CDCl_3_): δ = 143, 137.1, 128.8, 128.5, 127.7, 126.4, 126.2, 124.5, 121.9.

MS (EI): found: 186; calcd for C_12_H_10_S: 186.

**References (Supporting Information)**

1. Badone, D.; Baroni, M.; Cardamone, R.; Ielmini, A.; Guzzi, U. *J. Org. Chem.* **1997,** *62,* 7170–7173. doi:[10.1021/jo970439i](http://dx.doi.org/10.1021/jo970439i)
2. Fittig, R. *Justus Liebigs Ann. Chem.* **1862,** *121,* 361–365. doi:[10.1002/jlac.18621210308](http://dx.doi.org/10.1002/jlac.18621210308)
3. Gattermann, L. *Justus Liebigs Ann. Chem.* **1906,** *347,* 347–386. doi:[10.1002/jlac.19063470306](http://dx.doi.org/10.1002/jlac.19063470306)
4. Moore, L. R.; Shaughnessy, K. H. *Org. Lett.* **2004,** *6,* 225–228. doi:[10.1021/ol0360288](http://dx.doi.org/10.1021/ol0360288)
5. Wolfe, J. P.; Singer, R. A.; Yang, B. H.; Buchwald, S. L. *J. Am. Chem. Soc.* **1999,** *121,* 9550–9561. doi:[10.1021/ja992130h](http://dx.doi.org/10.1021/ja992130h)
6. Solodenko, W.; Mennecke, K.; Vogt, C.; Gruhl, S.; Kirschning, A. *Synthesis* **2006,** 1873–1881. doi:[10.1055/s-2006-942370](http://dx.doi.org/10.1055/s-2006-942370)
7. Hübner, H. *Ber. Dtsch. Chem. Ges.* **1908,** *41,* 482–487. doi:[10.1002/cber.19080410189](http://dx.doi.org/10.1002/cber.19080410189)
8. Williams, H. W. R.; Eichler, E.; Randall, W. C.; Rooney, C. S.; Cragoe, E. J., Jr.; Streeter, K. B.; Schwam, H.; Michelson, S. R.; Patchett, A. A.; Taub, D. *J. Med. Chem.* **1983,** *26,* 1196–1200. doi:[10.1021/jm00362a020](http://dx.doi.org/10.1021/jm00362a020)
9. Farina, V.; Krishnan, B.; Marshall, D. R.; Roth, G. P. *J. Org. Chem.* **1993,** *58,* 5434–5444. doi:[10.1021/jo00072a028](http://dx.doi.org/10.1021/jo00072a028)
10. Pfeiffer, P.; Sergiewskaja, S. *Ber. Dtsch. Chem. Ges.* **1911,** *44,* 1107–1112. doi:[10.1002/cber.191104401164](http://dx.doi.org/10.1002/cber.191104401164)
11. Ried, W.; Schäfer, D. P. *Chem. Ber.* **1970,** *103,* 2225–2233. doi:[10.1002/cber.19701030726](http://dx.doi.org/10.1002/cber.19701030726)
12. Michaelis, A.; Lange, H. *Ber. Dtsch. Chem. Ges.* **1875,** *8,* 1313–1316. doi:[10.1002/cber.187500802124](http://dx.doi.org/10.1002/cber.187500802124)
13. Kirilov, M.; Petrova, J.; Momchilova, S.; Galunski, B. *Chem. Ber.* **1976,** *109,* 1684–1693. doi:[10.1002/cber.19761090512](http://dx.doi.org/10.1002/cber.19761090512)
